# One-stage hip reconstruction in children with cerebral palsy: long-term results at skeletal maturity

**DOI:** 10.1007/s11832-014-0589-9

**Published:** 2014-05-06

**Authors:** Cindy Mallet, B. Ilharreborde, A. Presedo, A. Khairouni, K. Mazda, G. F. Penneçot

**Affiliations:** 1 Pediatric Orthopedic Department, Robert Debre University Hospital, Paris Diderot University, 48 bd Serurier, 75019 Paris, France; 2 Pediatric Orthopedic Institute, 34 rue Charam Achaykh, Casablanca, Morocco

**Keywords:** Spastic hip, Femoral osteotomy, Dega acetabuloplasty, Closed reduction

## Abstract

**Purpose:**

Hip subluxation is common in children with cerebral palsy (CP). Surgery is indicated in case of pain or progressive increase of Reimers index on radiographs. Peri-iliac osteotomy combined with femoral osteotomy is one of the numerous operative techniques available, but results at skeletal maturity remain unclear. The purpose of this radiological study was to report the long-term results of this procedure.

**Materials and methods:**

Twenty hips in 20 children were retrospectively evaluated at skeletal maturity. Mean age at surgery was 8.1 years and follow-up averaged 9.1 years. All patients underwent Dega acetabuloplasty, soft-tissue release and femoral-shortening varus derotation osteotomy without open reduction. Reimers index, acetabular angle (AA) and neck-shaft angle (NSA) were compared on preoperative, postoperative and latest follow-up radiographs.

**Results:**

Dega osteotomy significantly improved the AA and the correction remained stable at maturity. The NSA significantly decreased postoperatively (153°–115°), but recurrence of the valgus deformity (130°) of the proximal femur was observed at maturity. Consequently, Reimers index followed the same evolution. No case of osteonecrosis was reported but one hip dislocated and one subluxated during follow-up.

**Conclusion:**

Progressive recurrence of the valgus deformity of the proximal femur, attributable to adductors spasticity and gluteus medius weakness, led to a significant increase in the Reimers index. However, hip coverage remained >70 % at maturity in 90 % of the hips. This one-stage procedure without hip dislocation efficaciously corrected acetabulum dysplasia and successfully treated neurological hips in CP patients.

**Level of evidence:**

IV: retrospective study.

## Introduction

Progressive hip subluxation is one of the most common orthopaedic problems in patients with cerebral palsy (CP) [1]. The incidence ranges from 2.6 % to 75 % [2], and the risk of hip displacement is directly related to patient functional status [1]. Hip dislocation is caused by muscle imbalance, including spasticity of hip adductors and flexors and weakness of hip extensors and abductors, leading to osseous deformity with increased femoral anteversion, femoral neck valgisation, acetabular dysplasia and pelvic obliquity. Spastic hip displacement may be associated with complications such as difficulty with perineal care, poor sitting tolerance, and pain [3, 4]. Botulinum toxin injections and soft-tissue lengthening can be used in young patients in order to delay operative bony procedures [3, 4]. In older patients, one-stage reconstruction procedures associating femoral varus derotation and shortening osteotomy (VDRSO) and pelvic osteotomy are considered to be the most appropriate treatment [2–6]. In addition, some authors have advocated associating open reduction and capsulorrhaphy in case of hip dislocation [2–5] despite the risk of femoral head avascular necrosis (AVN).

In the literature, few studies have reported the results of this one-stage procedure with a long-term follow-up [4, 6]. However, skeletal modifications due to growth continue after surgical hip reduction in immature patients. The aim of this study was, therefore, to analyze the radiological outcomes at skeletal maturity of closed hip reduction by peri-iliac pelvic osteotomy associated with VDRSO in the treatment of hip displacement in children with CP.

## Materials and methods

### Patients

All patients who underwent one-stage hip reconstruction in our institution from 1994 to 2006 for neurological hip subluxation or dislocation related to CP, were retrospectively reviewed. All patients were skeletally immature at the time of surgery. To be eligible for the study, a minimum 2-year follow-up was required, and patients had to be at skeletal maturity (with closed triradiate cartilage) at the latest examination.

Indications for surgery were the indications described by Miller et al. [7]: (1) failure of soft-tissue release in children younger than eight years with Reimers migration percentage >40 % one year after surgery, (2) subluxated hips in children aged >8 years with Reimers migration percentage >40 %, (3) dislocated hips after <2 years from onset resulting in functional impairment regardless of age.

Collected data included: gender, type of involvement (diplegic or tetraplegic), functional status as described by the Growth Motor Function Classification System (GMFCS), age at surgery and age at follow-up.

### Surgical procedure

In all cases spastic hip was reduced by a one-stage procedure combining peri-iliac osteotomy, muscle lengthening (gracilis myotomy and adductor longus tenotomy), and intertrochanteric femoral VDRSO. Closed reduction was used in all cases, even if the hip was dislocated.

The first step was a combined gracilis and adductor longus release, performed through a transversal incision made in the groin.

Following this soft-tissue release, the femoral osteotomy was performed through a second incision in the lateral aspect of the proximal portion of the thigh. After exposing the femur, the lesser trochanter with the psoas insertion was removed from the metaphysis. The first femoral osteotomy extended from 1 cm below the growth plate of the greater trochanter to just above the position on the lesser trochanter. A derotation was then made to obtain approximately 30° of internal rotation of the hip at the end of the procedure. The second intertrochanteric osteotomy removed a trapezoidal-shaped piece of bone allowing 2–3 cm of femoral shortening and a decrease in neck-shaft angle (NSA) to 110°. A NSA of 110° was considered to be adequate to obtain a satisfactory reduction of the hip. Lower NSA (90° or 100°) could result in skin ulceration over the greater trochanter. The osteotomy was then fixed using a Maconor^®^ straight plate. At this stage of the procedure, an intraoperative radiograph was performed to verify that the hip was reduced.

The acetabuloplasty procedure that was performed was a Dega modified osteotomy (or San Diego osteotomy), as described by Mubarak et al. [3]. Through a Smith–Petersen approach, a curvilinear osteotomy was made 0.5–1 cm above the acetabular margin extending anteriorly and posteriorly through the sciatic notch. Under fluoroscopic guidance, it extended from the lateral cortex in the direction of the triradiate cartilage without crossing it. The osteotomy was progressively opened until maximum coverage of the femoral head was obtained. The bone removed from the femoral osteotomy was then reshaped and placed in the acetabular osteotomy site, as described by Sankar et al. [4]. The difference with the classical Dega osteotomy was that cuts were performed in the anterior and posterior edges of the pelvis to improve the posterior coverage of the femoral head. A bilateral spica cast was applied postoperatively for six weeks, with hips in extension, neutral rotation and 30° of abduction, in order to (1) decrease the postoperative pain, (2) lower the risk of osteotomy displacement and (3) maintain bilateral hips abduction. The cast was open in order to allow skin verification and prevention of pressure ulcers

### Radiological assessment

Anteroposterior radiographs of the pelvis were obtained preoperatively, postoperatively (within seven days) and at latest follow-up. The measured parameters were: Reimers migration index [8], the acetabular angle (AA) and the NSA. When the triradiate cartilage was open, the acetabular angle corresponded to the acetabular index [9] described as the angle between the Hilgenreiner line and a line drawn from the triradiate cartilage to the lateral edge of the acetabulum. At latest-follow-up, the triradiate cartilage was closed, the acetabular angle measured was the acetabular roof angle of Tonnis [10] (HTE angle) described as the angle between a line drawn from the acetabular background to the lateral edge of the acetabulum.

The NSA angle was measured on anteroposterior radiographs with the hip in extension, abduction and the patella pointing upwards as described in the literature [11]. As previously published [2], hip subluxation was defined as a migration index from 35 % to 89 %, while hip dislocation corresponded to a migration index ≥90 %.

After closure of the triradiate cartilage, follow-up radiographs were assessed by a senior orthopaedic surgeon, using the Melbourne Cerebral Palsy Hip Classification System (MCPHCS) [12]. Radiographs were also reviewed to determine the presence of osteonecrosis, joint incongruity or degenerative changes. At latest follow-up, spinal X-rays were also performed for scoliosis detection and to look for any pelvic obliquity.

The hip reconstruction was considered as a success if the MCPHCS was graded 1 or 2 (Reimers Index ≤15 %).

### Statistical analysis

After normality testing (Shapiro–Wilk test), a non-parametric matched-pairs Wilcoxon test, with significant *p* value <0.05, was used to compare preoperative, postoperative and last follow-up of radiological data. The course of the three radiological parameters (from the postoperative period to last follow-up) was analyzed according to age at surgery (after and before eight years) using an unpaired non-parametric Mann–Whitney test with a significant *p* value <0.05. All statistical analyses were conducted using SPSS version 12.0 (SPSS Inc., Chicago, IL, USA)

## Results

### Patients

A total of 30 patients were treated by this one-stage procedure for neurologic hip displacement from 1994 to 2006. Ten were excluded due to insufficient radiological data.

Twenty one-stage hip reconstructions were performed in those 20 patients (12 males and eight females). All children had spastic quadriplegia, with a functional status GMFCS at 4 in two patients, and at 5 in 18 patients. Eighteen hips were subluxated and two were dislocated for <2 years. Demographic data are summarized in Table 1. Age at the time of surgery averaged 8.1 ± 3.5 years (range 3.5–13.5 years). The mean follow-up was 9.1 ± 2.5 years (range 5.5–14.4 years), and age at final follow-up averaged 18.1 ± 2.4 years (range 14.5–22.4 years. Eleven patients (55 %) were operated before eight years of age.

**Table 1:** Demographic and radiological data

Patients	Sex	GMFCS status	Age at surgery (years)	Follow-up (years)	Acetabular angle (°)			Reimers index (%)			Neck-shaft angle (°)			Complications	MCPHSC score	Others surgical procedures
					Pre-op	Post-op	Last follow-up	Pre-op	Post-op	Last follow-up	Pre-op	Post-op	Last follow-up			
1	F	5	5.3	10.0	28	28	26	57	35	86	146	108	150	Hip dislocation	5	N
2	F	5	8.1	10.5	34	24	18	53	23	44	148	120	170	Hip subluxation	4	N
3	F	5	4.5	10.1	34	5	12	61	0	8	168	115	138	N	1	N
4	M	5	3.5	14.4	33	15	22	55	0	0	170	118	140	Contralateral hip subluxation	1	Contralateral hip one-stage reconstruction
5	F	5	6.2	6.2	30	10	8	65	0	0	160	130	135	N	1	N
6	M	5	6.5	6	38	15	8	58	0	0	166	138	130	N	1	N
7	F	5	5.7	9.1	32	22	20	50	15	25	150	118	120	N	3	N
8	F	5	12.2	7.8	27	8	10	47	0	17	150	108	130	N	3	N
9	F	4	9.6	11.5	35	15	20	68	13	0	150	105	130	N	1	Spine fusion
10	M	4	13.2	7.6	45	10	5	100	0	0	175	110	130	N	1	N
11	M	5	5.3	12.5	12	5	24	50	0	0	160	110	110	N	1	N
12	M	5	9.1	11.2	24	10	18	50	8	26	95	90	120	N	3	Spine fusion
13	M	5	5.0	10.2	31	5	17	100	0	0	160	130	125	N	1	N
14	M	5	7.9	6.7	24	14	13	31	0	26	138	82	92	N	3	N
15	M	5	4.5	11.5	28	6	12	52	0	20	150	117	130	Contralateral hip subluxation	3	Contralateral hip one-stage reconstruction
16	M	5	8.7	6.9	32	13	15	70	0	25	154	114	132	N	3	N
17	M	5	10.9	8.0	20	5	20	54	0	0	140	123	125	Contralateral hip subluxation	1	Spine fusion
18	F	5	10.1	5.5	35	15	18	70	0	0	150	100	110	Contralateral hip subluxation	1	N
19	M	5	12.9	7.8	30	16	14	60	0	15	170	142	148	N	2	N
20	M	5	13.5											Septic pseudarthrosis	6	Femoral head resection

### Radiological parameters

**Fig. 1:**
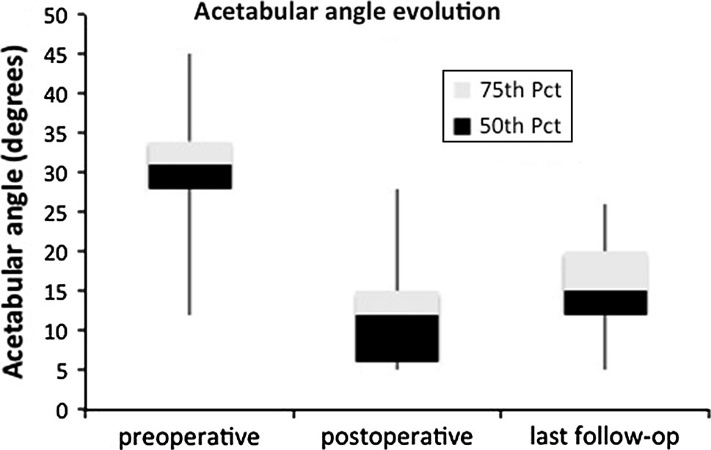
Evolution of acetabular angle at preoperative, postoperative and final evaluation. *Pct* percentile

**Fig. 2:**
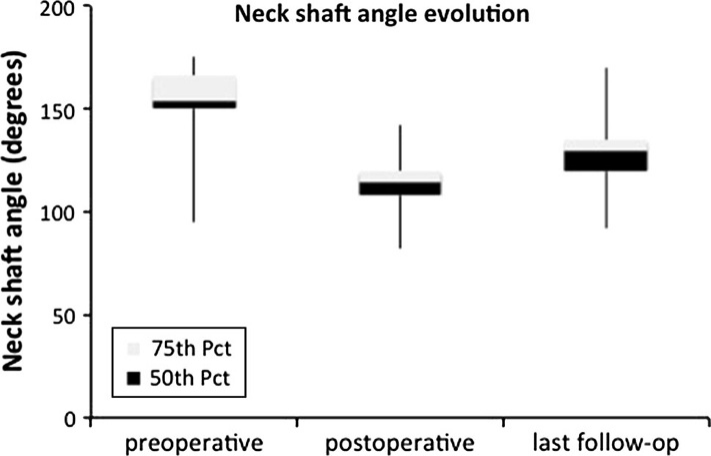
Evolution of neck-shaft angle at preoperative, postoperative and final evaluation. *Pct* percentile

**Fig. 3:**
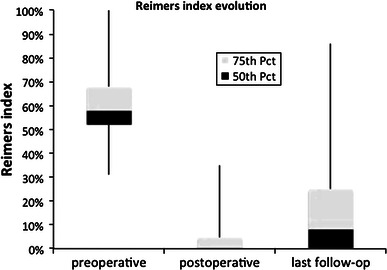
Evolution of migration percentage of Reimers index at preoperative, postoperative and final evaluation. *Pct* percentile

Radiological data are summarized in Table 1. The mean AA was significantly reduced after surgery (*p* < 0.05) and remained stable over time (*p* = 0.32) (Fig. 1). The NSA decreased significantly postoperatively (*p* < 0.05), but significant recurrence of the proximal femur valgus deformity was observed at latest-follow-up (*p* < 0.05) (Fig. 2). Consequently, the Reimers index followed the same course with significant reduction postoperatively (*p* < 0.05), and significant progression during follow-up (*p* = 0.05) (Fig. 3).

The parameters evolution was estimated by dividing the angles or Reimers progression by the time of follow-up, to get a sense of rate of their progression: the Reimers index increased by a mean of 1.1 % a year, the AA by a mean of 0.3° per year and the NSA by 1.5° a year.

**Fig. 4:**
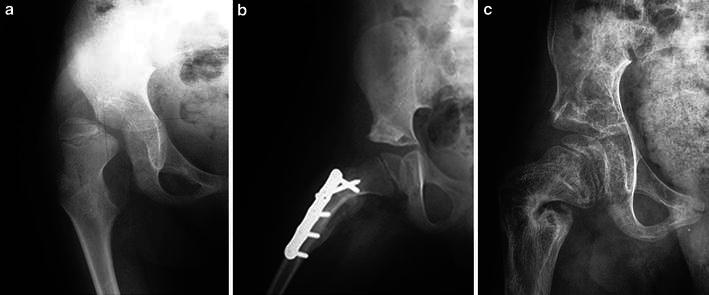
**a** Pre-operative X-rays of a 6-year-old boy with neurologic hip dislocation. **b** Postoperative result after Dega procedure and femoral shortening, varus and derotation osteotomy. **c** Long-term follow-up

**Fig. 5:**
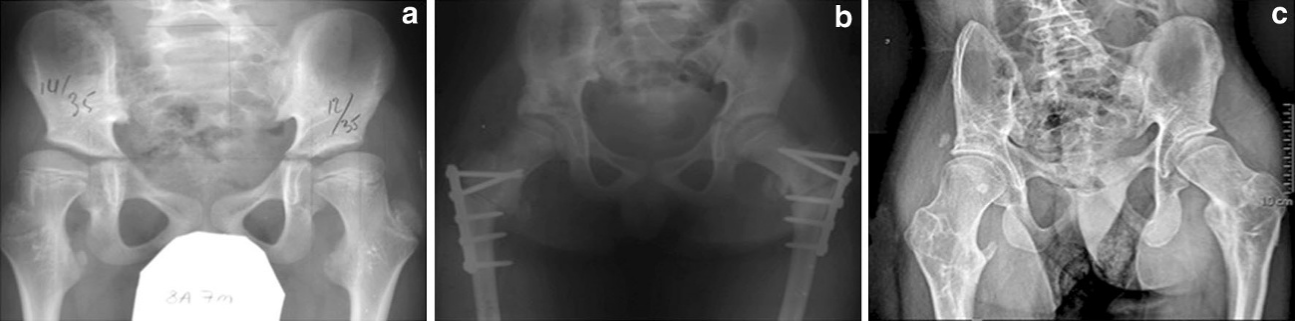
**a** Pre-operative X-ray of a 8-year-old boy with bilateral hip excentration. **b** Postoperative X-rays: *right hip*: Dega procedure and femoral shortening, varus and derotation osteotomy. *Left hip* femoral osteotomy alone. **c** Long-term follow-up

At latest examination, 17 hips (85 %) remained reduced (Figs. 4, 5).

No difference was found regarding the postoperative course of the Reimers index, the AA and the NSA between patients operated before and after eight years of age (Table 2).

**Table 2:** Neck shaft angle, acetabular angle and Reimers index evolution depending on the age

Neck shaft angle evolution (°)		Acetabular angle evolution (°)		Reimers index evolution (%)	
<8 years	>8 years	<8 years	>8 years	<8 years	>8 years
55	18	7	−6	8	21
22	18	7	2	0	25
13	25	6	8	20	19
−2	26	17	4	0	−13
0	10	24	−12	0	0
22	2	−2	15	51	0
2	40	−2	2	10	17
43	8	8	−2	28	15
5	−5	−2	−10	0	0
−8		−7		0	
10		−1		0	
p* = 0.45		p* = 0.15		p* = 0.08	

p*, *p* value. Mann–Whitney test

### Complications

No intraoperative or early postoperative complication was reported. Three months postoperatively, one patient (5 %) developed a proximal femoral septic non-union that required femoral head resection. At latest examination, one hip (5 %) showed an increased Reimers index of more than 30 %, and another hip (5 %) was dislocated. In these two patients (Table 1: patients 1 and 2), operated at the age of 8.1 and 5.3 years, respectively, the progression of the AA and the NSA overtime was analysed on different X-rays: the AA remained stable whereas the NSA tended to increase with time (patient 1: NSA increased from 108° postoperatively to 150° at latest follow-up and for patient 2 the NSA increased from 120° to 170°). These poor results were primarily attributable to the recurrence of the coxa valga deformity overtime. After an average delay of nine years following hip surgery, three patients (15 %) required spinal arthrodesis at maturity in order to correct a progressive scoliosis and prevent further progression of pelvic obliquity. Four patients (20 %) had a subluxation of the contralateral hip during follow-up, and two underwent the same one-stage procedure. The results of those two surgeries were not included in the present study because they were operated close to skeletal maturity.

There was no radiological evidence of AVN, osteoarthritis, or premature closure of the triradiate cartilage, and no pathological fracture of the femur was observed after cast removal.

## Discussion

In patients with spastic CP, hip reduction can be obtained by a combination of soft-tissue releases, VDRSO, and pelvic osteotomy, depending on the severity of the hip displacement and the age of the patient [2, 3]. The current study is the first one to report the results of a one-stage closed reconstruction strategy at skeletal maturity. Radiological results were satisfactory, with only 10 % of recurrence of subluxation or dislocation at skeletal maturity, and consistent with the results published in the literature, ranging from 7 % to 23 % [4–6, 13]. However, Sankar et al. [4] and Jozwiack et al. [5] systematically performed an open reduction and associated capsulorrhaphy, while McNerney et al. [13] recommended open reduction associated with capsulorrhaphy in case of Reimers >70 %. Their results are comparable to those described here. In the current study, closed reduction was chosen in all cases in order to avoid AVN, even in dislocated hips, and 100 % of the hips that were dislocated before the operation remained successfully reduced at latest follow-up. The recurrence of subluxation in one case and the dislocation observed in another in the present study were due to recurrence of coxa valga deformity of the proximal femur and open reduction would probably not have changed their outcome. In our experience, the only theoretical indication for open reduction is a hip dislocation for more than two years.

At skeletal maturity, 55 % (11/20) of the hips were considered as normal or near normal hips, 30 % (6/20) of the hips were dysplastic and 10 % (2/20) were either subluxated or dislocated. Those results are comparable to those previously reported by Dhawale et al. [6]. However, they reported that 68 % of the hips needed further surgery in their study while no hips required revision surgery in the present series.

### Results of femoral osteotomy

Progressive coxa valga recurrence of the upper femoral extremity during growth was observed in this study (*p* < 0.001), progressively altering the Reimers migration percentage. This finding is consistent with previous literature, and can be explained by (1) imbalance between the spasticity of psoas and adductor muscles and (2) gluteus medius muscle weakness [5, 14, 15]. Nevertheless, the gradual migration of the femoral head was moderate [16], with 85 % of the hips maintaining a Reimers index <30 % at skeletal maturity. Noonan et al*.* [2] and Khalife et al. [17] reported that age at surgery <6 years was a prognosis factor for stabilization, and that young age was associated with better radiological outcomes because of potential bone remodelling. In our series, recurrent coxa valga (*p* = 0.45) and course of Reimers index (*p* = 0.08) were not influenced by the age at intervention.

### Choice of pelvic osteotomy

Different pelvic osteotomies have been described in to correct the acetabulum dysplasia. Innominate Salter osteotomy and triple osteotomy have given unsatisfactory results for the treatment of spastic subluxation of the hip [18, 19]. Pelvic innominate osteotomy increases the anterolateral coverage of the femoral head and decreases the posterior coverage, which was the reason why Salter et al. [19] advised against its use in neurologic hip subluxation. The literature remains controversial regarding the analysis of the acetabular deficiency. Zimmermann and Sturm [20] and Gugenheim et al*.* [21] reported the acetabulum deficiency to be anterior on 2-dimensional CT, whereas Buckley et al*.* [22] reported it to be posterior on 2-dimensional CT. Recently, Chang et al*.* [23] used 3D CT and confirmed that the acetabulum deficiency was more anterior. The acetabulum of CP patients with hip subluxation or dislocation is usually ovoid and elongated [4]. For all these reasons, the Dega modified acetabuloplasty seems to be the most appropriate osteotomy. This procedure allows a global femoral head coverage that can be adjusted intraoperatively by the position of the bone graft. The main advantage is the posterior correction obtained, without sacrificing the anterior coverage. Results of our study confirm that Dega pelvic osteotomy is not only efficient, but also stable over time, since the AA remained unchanged after nine years of follow-up.

One of the strengths of this study was the follow-up at skeletal maturity. This demonstrated that correction of the acetabular dysplasia by the Dega acetabuloplasty remained stable but that coxa valga deformity of the femur recurred. We could not determine whether the NSA stabilized after closure of the triradiate cartilage, because once the osteotomy had healed, the patients were only reviewed every two years, and intermediate X-rays were not necessarily obtained between the time of the closure of the triradiate cartilage and the last follow-up. Previous literature [6, 24] has also failed to be conclusive on this point. However, in those children with CP, combination of growth, muscle imbalance and spasticity was held to be responsible for hip dysplasia and recurrent coxa valga [1, 24, 25]. Consequently, skeletal maturity would stop this trend and stabilize the hip. Noonan et al. [25] reported that in adults with CP, the main hip problems are linked to pain due to windswept, spasticity with limitation of abduction, flexion contractures and osteoarthritis. According to those authors, hip displacement was not associated with hip pain.

### Management of the contralateral hip

The management of the contralateral hip in patients with unilateral spastic hip displacement remains controversial. In the literature, the rate of dislocation or subluxation of the contralateral hip in CP ranges from 6 % to 74 % [2, 13, 24, 26]. In the present study, the rate of contralateral hip subluxation (20 %) seemed low. One explanation could be that, at the time of surgery, we performed contralateral soft-tissue lengthening (gracilis and longus adductors release) in case of hip abduction <30°. That might have helped to stabilize the hip, once the other hip had been reconstructed. For Shukla et al. [26], predictors of contralateral hip subluxation are lack of contralateral soft-tissue release, pelvic obliquity angle, and larger initial contralateral hip femoral migration index >25 %. Some authors [7, 23] stated that bilateral hip surgery in non-ambulatory quadriplegic patients should be considered to maintain symmetry and pelvic alignment, even if the contralateral hip appears normal. However, Dhawale et al. [6] demonstrated that hip reconstruction was more difficult and more likely to fail after an unsuccessful VDRO than after soft-tissue release. For this reason, in our institution, we chose a more conservative strategy with soft-tissue lengthening performed in case of hip abduction <30°, but no preventive bony procedure in the hips that were normal on the X-rays. We believed that the contralateral hip should be closely followed until skeletal maturity to permit a one stage-reconstruction procedure if required as quickly as possible.

### Complications

Avascular necrosis is a potential serious complication after hip reduction, and no such case occurred in the present study. This low rate can be explained by (1) the relatively great extent of femoral shortening (2–3 cm), leading to gentle reduction without cephalic vessel tension and (2) the closed reduction that avoided vascular damage. In addition, McNerney et al. [13] also noted that vessel injury could occur in the groin during ilio-psoas lengthening, which is why psoas insertion was detached at the level of the lesser trochanter.

The Dega osteotomy was carefully guided under fluoroscopy, in order to visualize the direction of the bone chisel, and no premature closure of the triradiate cartilage was reported.

### Limitations

One of the main limitations of our study is that it was strictly radiological. Further functional and clinical assessments remain necessary. Pelvic obliquity would have brought further interesting information, however this parameter was impossible to measure on many radiographs in this retrospective study. In addition, inter-examiner and intra-examiner reproducibility of the radiological measurements were not evaluated. Moreover, the number of patients included was relatively small. Nonetheless, the follow-up was one of the longest reported in the literature, and all patients were reviewed at skeletal maturity.

In conclusion, the one-stage closed reconstruction procedure, combining Dega modified acetabuloplasty, VDRSO, and soft tissue lengthening, provided long-lasting satisfactory radiological outcomes in CP patients. However, progressive recurrence of the proximal femur deformity (coxa valga) can be observed in young patients, requiring long-term follow-up.
